# Emotional Exhaustion, Moral Sensitivity, and Happiness Among Nurses in Cardiac Wards: A Multicenter Cross‐Sectional Study

**DOI:** 10.1155/jonm/6483265

**Published:** 2026-10-06

**Authors:** Safoura Dorri, Seyed Ali Rasooli

**Affiliations:** ^1^ Nursing and Midwifery Care Research Center Isfahan University of Medical Sciences Isfahan Iran mui.ac.ir; ^2^ Department of Emergency Medicine School of Nursing and Midwifery Chronic Disease (Home Care) Research Center Institute of Cancer Hamadan University of Medical Sciences Hamadan Iran umsha.ac.ir

**Keywords:** burnout, cardiovascular nursing, happiness, nursing, practical nurses, professional ethics

## Abstract

**Aim(s):**

To examine associations of emotional exhaustion and moral sensitivity with happiness among nurses in cardiac wards.

**Background:**

Cardiac nursing involves sustained emotional demands and ethical challenges, yet their relative associations with happiness are unclear.

**Method(s):**

This multicenter cross‐sectional study randomly sampled 220 nurses from four Iranian teaching hospitals. Validated questionnaires assessed happiness, moral sensitivity, and emotional exhaustion. Pearson correlations were primary, with Spearman sensitivity analyses. HC3‐robust multivariable linear regression estimated adjusted associations.

**Results:**

Happiness was inversely correlated with emotional exhaustion (*r* = −0.409, 95% CI −0.515 to −0.291) and moral sensitivity (*r* = −0.245, 95% CI −0.374 to −0.106). In the complete‐case model (*n* = 181), emotional exhaustion remained clearly associated with lower happiness (*B* = −0.184, 95% CI −0.262 to −0.107), whereas the adjusted moral sensitivity association was smaller and less precise (*B* = −0.069, 95% CI −0.140 to 0.002). Sensitivity analyses supported the stability of the emotional exhaustion estimate.

**Conclusion(s):**

Emotional exhaustion showed the stronger and more consistent association with happiness; however, findings should be interpreted considering the modest reliability of the happiness measure and the cross‐sectional design, which limits causal inference.

**Implications for Nursing Management:**

Nurse managers should address modifiable work conditions and recovery opportunities while preserving organizational support for ethical practice.

## 1. Background

Nurse well‐being is a workforce and management concern because persistent occupational strain can affect retention, work functioning, and the quality and safety of care. Emotional exhaustion, the depletion component of burnout, is particularly relevant in high‐acuity settings where nurses must sustain attention, emotional regulation, and complex interpersonal work over repeated shifts. Burnout among nurses has been associated with adverse nurse and patient‐related outcomes, including poorer safety, quality, and satisfaction indicators [1]. Cardiac wards may intensify these demands because nurses frequently manage rapid deterioration, invasive treatments, symptom instability, end‐of‐life decisions, and distressed families while maintaining continuous surveillance and timely clinical responses. These conditions make emotional exhaustion relevant not only as an individual psychological state but also as a potential signal of how work is organized and supported.

Moral sensitivity represents a different dimension of nursing work. It reflects the capacity to recognize ethically salient features of care, understand patients’ vulnerability and values, anticipate consequences, and respond to the moral dimensions of clinical decisions. Studies in nursing have linked moral sensitivity with caring and ethical practice [2, 3]. In cardiac and other high‐acuity settings, ethical concerns may arise around urgent treatment decisions, competing priorities, end‐of‐life care, communication with families, and discrepancies between ideal and feasible care. Recognizing such concerns does not necessarily mean that nurses have sufficient resources or authority to resolve them. The well‐being implications of moral sensitivity may therefore depend on contextual factors such as ethical climate, staffing, workload, leadership, and access to organizational support.

Happiness is a positive component of occupational well‐being and may reflect how nurses experience their professional and personal resources within the work environment. Previous studies have associated nurses’ happiness with work environment, job satisfaction, quality of working life, and professional functioning [4–6]. Iranian evidence has also reported an inverse relationship between happiness and burnout [7]. Emotional exhaustion and moral sensitivity, however, represent conceptually distinct experiences: Emotional exhaustion reflects the depletion of occupational emotional resources, whereas moral sensitivity reflects ethical awareness and professional responsiveness. Examining them simultaneously may help distinguish whether lower happiness is more closely associated with emotional depletion, ethical sensitivity, or both.

Clarifying these relationships has practical relevance for nursing management. Existing studies have often examined happiness in relation to work environment, job satisfaction, or burnout, and moral sensitivity in relation to ethical practice or caring outcomes, but less is known about their simultaneous associations in cardiac nursing. If emotional depletion is more strongly related to nurses’ well‐being than ethical sensitivity after relevant covariates are considered, managerial responses should focus on modifiable work conditions and recovery resources without framing moral sensitivity as a problem to be reduced. This study therefore examined emotional exhaustion, moral sensitivity, and happiness among nurses working in cardiac wards and assessed the adjusted associations of emotional exhaustion and total moral sensitivity with happiness.

## 2. Methods

### 2.1. Study Design and Setting

A multicenter cross‐sectional study was conducted from September to November 2024 in four teaching hospitals affiliated with Isfahan University of Medical Sciences in Isfahan, Iran. Recruitment covered internal cardiac wards, cardiac surgery wards, and special cardiac care units, which differed in patient acuity and work organization but all involved direct cardiovascular nursing care. Reporting was guided by the Strengthening the Reporting of Observational Studies in Epidemiology (STROBE) recommendations for cross‐sectional studies [8].

### 2.2. Participants, Sample Size, and Sampling

Hospital nursing rosters were screened before random selection. Eligible sampling frames included registered nurses working in internal cardiac wards, cardiac surgery wards, or special cardiac care units who had at least 1 year of clinical experience. Nurses reporting a major stressful life event during the preceding 6 months, a diagnosed psychological disorder, or use of psychotropic medication or narcotic substances were not included in the eligible sampling frames. Written informed consent was obtained from selected nurses before questionnaire completion. The retained study records document the final enrolled cohort but do not permit reliable retrospective reconstruction of the total number screened or excluded at each pre‐enrollment criterion; these counts were therefore not estimated retrospectively.

The sample size was estimated a priori in G∗Power 3.1 for the primary two‐sided correlation objective. A conservative small‐to‐moderate expected correlation of *r* = 0.20, alpha = 0.05, and 80% power required 194 analyzable participants. A pragmatic 10% allowance for potentially incomplete or unusable questionnaires was applied prospectively (194/0.90 = 215.6), yielding a recruitment target of 216. Recruitment slightly exceeded this target, with 220 nurses enrolled. The calculation addressed the primary correlation objective rather than the subsequent explanatory multivariable model.

Within each hospital, participants were selected from the eligible sampling frame using a random‐number procedure, with the number selected reflecting the available eligible nursing workforce. Recruitment was conducted across morning, evening, and night shifts. Selected nurses received information about the study purpose, confidentiality, voluntariness, and approximate completion time before providing written informed consent.

### 2.3. Measures

Data collection included a sociodemographic and occupational characteristics form, the short Oxford Happiness Questionnaire (OHQ), the Moral Sensitivity Questionnaire, and the Maslach–Jackson Emotional Exhaustion scale. The study‐developed characteristics form included age, sex, marital status, education, employment status, clinical experience, hospital, cardiac‐unit type, income, housing, second job, physical activity, leisure time, night shifts, perceived health, interest in nursing and the work environment, and conflict with others. Ten faculty members with relevant expertise reviewed its face and content validity.

Happiness was assessed using the 8‐item short form of the OHQ (OHQ‐SF). Items are rated from 1 (Strongly Disagree) to 6 (Strongly Agree); Items 1, 4, and 8 are reverse‐scored before summation, yielding a total score from 8 to 48, with higher scores indicating greater happiness [9]. The validated Persian short form was used, and no new translation was undertaken for this study. Previous evaluation of the Persian OHQ‐SF reported Cronbach’s alpha of approximately 0.71 [10]. Internal consistency was re‐estimated in the present sample.

Moral sensitivity was assessed using the validated 25‐item Persian Moral Sensitivity Questionnaire. Items are scored from 0 to 4 and summed to a total score from 0 to 100; higher scores indicate greater moral sensitivity. The six predefined subscales are respect for patient autonomy, awareness of communication with patients, professional knowledge, experiencing ethical problems and dilemmas, applying ethical concepts in decision‐making, and honesty and benevolence. The Persian adaptation used translation and back‐translation, expert review of face and content validity, and pilot reliability assessment; previous Iranian research reported total‐scale Cronbach’s alpha around 0.81 [11]. Additional Iranian studies have supported use of the 25‐item form [12, 13]. Internal consistency of the total scale was estimated again in the current sample.

Emotional exhaustion was measured using the validated Persian version of the nine‐item Maslach–Jackson Emotional Exhaustion scale. In the Persian version administered in this study, each item was scored from 1 to 6, yielding a total score from 9 to 54; higher scores indicate greater emotional exhaustion. The scale was analyzed continuously, and no categorical diagnosis of burnout was inferred. Previous Iranian research reported high internal consistency and construct‐validity evidence for the emotional exhaustion measure [14]. Internal consistency was re‐estimated in the present sample.

### 2.4. Statistical Analysis

Primary analyses were conducted in IBM SPSS Statistics Version 26. Before analysis, data were checked for completeness, coding consistency, plausible ranges, reverse scoring, and agreement between item‐level responses and calculated total scores. Descriptive analyses used available observations, correlations used pairwise‐valid observations, and the primary multivariable model used complete cases. Analytic denominators are reported for all principal analyses. Internal consistency of the three total scales was assessed using Cronbach’s alpha, and distributions were examined descriptively and with the Shapiro–Wilk test.

Exploratory differences in happiness across sociodemographic, occupational, lifestyle, and work‐related characteristics were examined using independent‐samples *t* tests or one‐way analysis of variance, with Welch alternatives when variance homogeneity was not supported. Cohen’s *d* and eta‐squared quantified effect size. Because 18 omnibus comparisons were conducted for the same outcome, Holm adjustment was applied across the complete family of 18 tests to control the familywise error rate. These analyses were treated as exploratory rather than confirmatory.

Pearson correlations were the primary estimates for relationships among happiness, emotional exhaustion, and total moral sensitivity, with Spearman correlations as distributional sensitivity analyses. All correlation coefficients are reported with 95% confidence intervals. Associations between happiness and all six predefined Moral Sensitivity Questionnaire subscales were examined as a separate exploratory secondary family; Holm adjustment was applied across the six subscale correlations. “Predefined” refers to the established instrument structure and does not imply the prospective registration of these secondary correlation analyses.

A multivariable linear regression model examined happiness as the dependent variable. Emotional exhaustion and total moral sensitivity were the principal explanatory variables; age, sex, physical activity, income, and interpersonal conflict were retained as conceptually relevant covariates in the original explanatory model. Emotional exhaustion, moral sensitivity, and age were grand‐mean centered before estimation to provide an interpretable intercept. HC3 heteroscedasticity‐consistent standard errors were used. Residual distribution, heteroscedasticity, autocorrelation, multicollinearity, and influence were assessed using the Shapiro–Wilk test, Breusch–Pagan tests, Durbin–Watson statistic, variance inflation factors, externally studentized residuals, and Cook’s distance. Square‐root, logarithmic, and Box‐Cox outcome transformations were examined as sensitivity analyses; no transformation provided a consistent diagnostic advantage while retaining interpretation on the original happiness scale.

Sensitivity analyses added during revision examined incomplete data and hospital imbalance. Because missing scale totals arose principally from item nonresponse, donor‐based predictive mean matching was conducted at item level across 50 imputed datasets; scale totals were recalculated within each dataset, and estimates were pooled using Rubin’s rules. Inverse‐probability weighting provided an additional missing‐data sensitivity analysis. Because only four hospitals were available, hospital was entered as a fixed categorical effect rather than relying on random‐effect variance estimation or conventional cluster‐robust inference from four clusters. Leave‐one‐hospital‐out analyses assessed whether the dominant hospital drove the principal association. Revision sensitivity analyses were implemented in Python 3.13.5 using SciPy 1.17.0 and statsmodels 0.14.6. All tests were two‐sided, with an emphasis on effect estimates and 95% confidence intervals rather than binary interpretation around *p* = 0.05.

### 2.5. Ethical Considerations

The study received approval from the Ethics Committee of Isfahan University of Medical Sciences (IR.MUI.NUREMA.REC.1403.074) before data collection and was conducted in accordance with the Declaration of Helsinki. Participants received information about study procedures, voluntariness, confidentiality, and the right to withdraw without consequence. Written informed consent was obtained before questionnaire completion. Questionnaires were anonymous, and study data were used for research purposes only.

## 3. Results

### 3.1. Participant Characteristics

All 220 enrolled nurses were retained in the descriptive sample. Mean age was 32.43 years (SD = 6.84; median 31, IQR 27–37; range 22–52), and 72.3% were women. Most participants were married (62.3%) and held a bachelor’s degree (88.2%). Participants represented four teaching hospitals and three cardiac‐unit categories; hospital participation was uneven, with 69.5% recruited from one hospital. Additional characteristics are shown in Table 1 and Supporting Table S1.

**TABLE 1:** Sociodemographic, occupational, and work‐related characteristics of nurses working in cardiac wards.

Characteristic	Category/statistic	Result	Missing, *n*
Panel A. Sociodemographic characteristics			
Age (years)	Mean ± SD	32.43 ± 6.84	5
Age (years)	Median (IQR), range	31 (27–37), 22–52	—
Age group (years)	20–29	86/215 (40.0)	5
	30–39	92/215 (42.8)	—
	40–49	33/215 (15.3)	—
	≥ 50	4/215 (1.9)	—
Sex	Female	159/220 (72.3)	0
	Male	61/220 (27.7)	—
Marital status	Married	137/220 (62.3)	0
	Single	83/220 (37.7)	—
Education level	Bachelor’s degree	194/220 (88.2)	0
	Master’s degree	26/220 (11.8)	—
Panel B. Occupational and work‐related characteristics			
Employment status	Obligatory service	29/218 (13.3)	2
	Contractual, temporary, or corporate	121/218 (55.5)	—
	Permanent	68/218 (31.2)	—
Clinical work experience (months)	< 60	85/220 (38.6)	0
	60–119	68/220 (30.9)	—
	120–179	37/220 (16.8)	—
	180–239	15/220 (6.8)	—
	240–312	9/220 (4.1)	—
	> 312	6/220 (2.7)	—
Participating hospital	Alzahra Hospital	26/220 (11.8)	0
	Feiz Hospital	10/220 (4.5)	—
	Noor Hospital	31/220 (14.1)	—
	Chamran Hospital	153/220 (69.5)	—
Cardiac ward type	Internal cardiac ward	74/220 (33.6)	0
	Cardiac surgery ward	50/220 (22.7)	—
	Special cardiac care units	96/220 (43.6)	—
Monthly income, million Tomans	< 10	18/220 (8.2)	0
	10–15	187/220 (85.0)	—
	15–20	6/220 (2.7)	—
	> 20	9/220 (4.1)	—
Physical activity	Yes	81/220 (36.8)	0
	No	139/220 (63.2)	—
Night shifts per month	0–4	62/218 (28.4)	2
	4–8	75/218 (34.4)	—
	> 8	81/218 (37.2)	—
Conflict with others	High	18/217 (8.3)	3
	Moderate	72/217 (33.2)	—
	Low	127/217 (58.5)	—

*Note:* Data are presented as mean ± SD, median (IQR), range, or *n*/valid *N* (%), as appropriate. Percentages were calculated using valid denominators. IQR, interquartile range. An em dash indicates that the missing count is reported on the first row of the corresponding characteristic.

Abbreviation: SD, standard deviation.

Partial item nonresponse did not result in study‐level exclusion. Valid total scores were available for 216 participants for happiness, 195 for moral sensitivity, and 218 for emotional exhaustion. The complete‐case regression included 181 participants. Each of the 39 participants not included in that model was missing exactly one model variable: moral sensitivity (*n* = 25), age (*n* = 5), happiness (*n* = 4), interpersonal conflict (*n* = 3), or emotional exhaustion (*n* = 2). Missingness was associated with some observed characteristics, including hospital and physical activity; missing completely at random was therefore not assumed. Detailed patterns are presented in Supporting Table S8.

### 3.2. Study Variables and Reliability

Mean scores were 34.48 (SD = 5.82; 95% CI 33.70–35.26; *n* = 216) for happiness, 60.17 (SD = 13.04; 95% CI 58.33–62.01; *n* = 195) for total moral sensitivity, and 36.53 (SD = 10.90; 95% CI 35.07–37.98; *n* = 218) for emotional exhaustion. Cronbach’s alpha was 0.652, 0.858, and 0.936, respectively. Internal consistency of the happiness measure was therefore modest; deleting any individual happiness item did not materially improve alpha (maximum approximately 0.66), so the validated eight‐item scoring structure was retained. Full descriptive results are shown in Table 2; moral sensitivity subscale results, including percentage‐of‐maximum‐possible scores, are reported in Supporting Table S2.

**TABLE 2:** Descriptive statistics, internal consistency, and distributional properties of the main study variables.

Variable	Possible score range	*n*	Mean ± SD (95% CI)	Median (IQR)	Observed range	Cronbach’s *α*	Shapiro–Wilk *p*
Happiness	8–48	216	34.48 ± 5.82 (33.70–35.26)	35 (31–39)	21–46	0.652	0.001
Moral sensitivity, total	0–100	195	60.17 ± 13.04 (58.33–62.01)	58 (53–67)	13–100	0.858	< 0.001
Emotional exhaustion	9–54	218	36.53 ± 10.90 (35.07–37.98)	36 (28–45)	15–54	0.936	< 0.001

*Note:* IQR, interquartile range. Higher scores indicate higher levels of the corresponding construct. Cronbach’s alpha was calculated for the total scales. Shapiro–Wilk tests are descriptive diagnostics of distributional departure; distributional concerns were additionally addressed using rank‐based sensitivity analyses.

Abbreviations: CI, confidence interval; SD, standard deviation.

### 3.3. Exploratory Group Comparisons

Several characteristics showed nominal unadjusted differences in happiness, but none of the 18 exploratory omnibus comparisons remained statistically significant after Holm adjustment across the complete family of tests. The smallest Holm‐adjusted *p* values were 0.078 for income, 0.100 for interpersonal conflict, and 0.130 for physical activity. Nominal findings for clinical experience and income were also highly sensitive to categories containing only six participants: After excluding the > 312‐month experience group, *p* changed from 0.033 to 0.369, and after excluding the 15–20‐million‐Toman income group, Welch *p* changed from 0.004 to 0.885. These analyses were therefore treated as descriptive and hypothesis‐generating (Supporting Tables S4–S5).

### 3.4. Correlations

Correlation analyses among happiness, emotional exhaustion, and moral sensitivity are presented in Table 3. Happiness was inversely correlated with emotional exhaustion (*n* = 214; Pearson *r* = −0.409, 95% CI −0.515 to −0.291; *p* < 0.001) and total moral sensitivity (*n* = 191; *r* = −0.245, 95% CI −0.374 to −0.106; *p* < 0.001). Spearman sensitivity analyses were directionally similar. The happiness‐moral sensitivity association was also preserved after item‐level imputation (pooled *r* = −0.216, 95% CI −0.338 to −0.086; *p* = 0.001). The Pearson correlation between total moral sensitivity and emotional exhaustion was weak (*n* = 193; *r* = 0.133, 95% CI −0.008 to 0.269; *p* = 0.065), while the Spearman estimate was small and positive (rho = 0.152; *p* = 0.034), supporting cautious interpretation of this relationship. After Holm adjustment across the six moral sensitivity subscales, honesty and benevolence was the only subscale whose association with happiness remained statistically supported; this secondary result is hypothesis‐generating (Supporting Table S3).

**TABLE 3:** Correlations among emotional exhaustion, moral sensitivity, and happiness.

Relationship	*n*	Pearson *r*	95% CI	*p*	Spearman rho	Spearman *p*
Happiness × emotional exhaustion	214	−0.409	−0.515 to −0.291	< 0.001	−0.413	< 0.001
Happiness × moral sensitivity, total	191	−0.245	−0.374 to −0.106	< 0.001	−0.310	< 0.001
Moral sensitivity, total × emotional exhaustion	193	0.133	−0.008 to 0.269	0.065	0.152	0.034

*Note:* Pearson correlation coefficients were the primary estimates, and Spearman rank correlations were sensitivity analyses. All correlations are cross‐sectional and should not be interpreted as indicating temporal or causal relationships.

Abbreviation: CI, confidence interval.

### 3.5. Multivariable Analysis

The primary complete‐case regression included 181 participants and explained 26.4% of the observed variance in happiness (*R* ^2^ *= *0.264; adjusted *R* ^2^ = 0.230; *F* = 7.73; *p* < 0.001). Emotional exhaustion remained clearly associated with lower happiness after adjustment (*B* = −0.184, HC3 SE = 0.039, 95% CI −0.262 to −0.107; standardized beta = −0.354). The adjusted coefficient for total moral sensitivity was smaller and less precise (*B* = −0.069, HC3 SE = 0.036, 95% CI −0.140 to 0.002; standardized beta = −0.152). Age, sex, physical activity, income, and conflict indicators were estimated imprecisely after adjustment. Full coefficients are shown in Table 4.

**TABLE 4:** Multivariable linear regression model for factors associated with happiness among nurses working in cardiac wards.

Predictor	*B*	Robust SE (HC3)	95% CI	*t*	*p*	Standardized *β*	VIF
Constant	33.290	1.820	29.698 to 36.881	18.30	< 0.001	—	—
Emotional exhaustion	−0.184	0.039	−0.262 to −0.107	−4.68	< 0.001	−0.354	1.14
Moral sensitivity, total	−0.069	0.036	−0.140 to 0.002	−1.92	0.056	−0.152	1.09
Age (years)	0.038	0.048	−0.056 to 0.132	0.80	0.427	0.044	1.03
Male sex	1.340	0.995	−0.623 to 3.304	1.35	0.180	—	1.48
Physical activity, yes	0.880	1.060	−1.212 to 2.973	0.83	0.408	—	1.39
Income > 15 million Tomans	2.805	1.553	−0.261 to 5.871	1.81	0.073	—	1.15
Moderate conflict with others	−0.542	1.839	−4.171 to 3.088	−0.29	0.769	—	3.89
Low conflict with others	0.988	1.845	−2.653 to 4.629	0.54	0.593	—	4.05

*Note: B*, unstandardized coefficient; HC3, heteroscedasticity‐consistent standard error type 3. Dependent variable: happiness. Emotional exhaustion, total moral sensitivity, and age were grand‐mean centered before model estimation; centering changes the intercept but not slopes, standard errors, confidence intervals, *p* values, or model fit. Reference categories: female sex, no physical activity, income ≤ 15 million Tomans, and high conflict. Standardized beta is shown for continuous predictors. Model *n* = 181; *R* ^2^ = 0.264; adjusted *R* ^2^ = 0.230; *F* = 7.73; *p* < 0.001.

Abbreviations: CI, confidence interval; SE, standard error; VIF, variance inflation factor.

The emotional exhaustion estimate was highly stable across sensitivity analyses: *B* = −0.180 (95% CI −0.258 to −0.102) with hospital fixed effects, *B* = −0.191 (95% CI −0.265 to −0.117) after item‐level multiple imputation, *B* = −0.186 (95% CI −0.260 to −0.111) after combined imputation and hospital adjustment, and *B* = −0.189 (95% CI −0.269 to −0.109) with inverse‐probability weighting. Moral sensitivity estimates remained generally inverse but closer to the null boundary across the principal imputation and hospital‐adjusted analyses. Hospital adjustment increased *R* ^2^ only from 0.264 to 0.269, and hospital indicators were not jointly associated with happiness (robust *p* = 0.777). Emotional exhaustion estimates ranged from −0.177 to −0.207 when each hospital was omitted in turn; after complete exclusion of the hospital contributing 69.5% of the sample, B was −0.207 (95% CI −0.341 to −0.073). These results are summarized in Supporting Table S7.

Residuals showed a modest departure from normality (Shapiro–Wilk *W* = 0.971, *p* < 0.001; skewness = −0.53; excess kurtosis = 0.13), and Breusch–Pagan results were borderline for heteroscedasticity. No externally studentized residual exceeded an absolute value of 3, and the maximum Cook’s distance was 0.043. Alternative transformations did not provide a consistent overall diagnostic improvement; HC3 inference on the original happiness scale was therefore retained. Expanded diagnostics are reported in Supporting Table S6.

## 4. Discussion

This study examined two conceptually distinct correlates of happiness among nurses working in cardiac wards: emotional exhaustion as occupational emotional depletion and moral sensitivity as an ethical professional capacity. Both were inversely correlated with happiness in bivariate analyses. After covariate adjustment, however, the emotional exhaustion estimate remained comparatively large and precise, whereas the moral sensitivity estimate was smaller and its confidence interval narrowly included zero. This difference should not be reduced to a binary significant‐versus‐nonsignificant interpretation. Across missing‐data, hospital, and model‐specification sensitivity analyses, emotional exhaustion remained highly stable, whereas the moral sensitivity estimate was smaller and its statistical evidence was more sensitive to analytic approach. The cross‐sectional design cannot establish temporal ordering or causality.

The magnitude and consistency of the emotional exhaustion association are compatible with occupational stress frameworks. The job demands–resources model proposes that sustained job demands contribute particularly to exhaustion when adequate resources are unavailable [15], while Conservation of Resources theory conceptualizes stress as a process of threatened or depleted valued resources [16]. Cardiac nursing combines high acuity, rapid prioritization, repeated exposure to suffering, family distress, and ethically complex decisions. The present study did not measure the organizational mechanisms producing exhaustion, but the stability of the association across multiple analyses makes emotional exhaustion a credible workforce indicator for further management‐focused investigation. Recent Journal of Nursing Management research similarly links stress, well‐being, and burnout to workforce outcomes [17].

The moral sensitivity findings require a different interpretation. Moral sensitivity is a desirable professional capacity that enables nurses to recognize ethically salient features of care, patients’ values and vulnerability, and situations requiring ethical attention; it should not be framed as a harmful exposure. Its inverse bivariate relationship with happiness became smaller and less precise after emotional exhaustion and other covariates were considered. One plausible explanation is that ethical awareness may become emotionally demanding when nurses have insufficient authority, resources, communication channels, or organizational support to act on what they perceive as ethically appropriate. Moral ‐distress research is compatible with this possibility [18, 19], but the mechanism was not tested because moral distress, ethical climate, staffing adequacy, workload, and organizational support were not measured. The present data therefore support neither reducing moral sensitivity nor concluding that moral sensitivity independently lowers happiness.

Exploratory demographic and occupational comparisons did not remain statistically supported after familywise Holm adjustment and should not drive managerial conclusions. The honesty‐and‐benevolence subscale result also arose from secondary analyses and should remain hypothesis‐generating. In contrast, the emotional exhaustion association was supported by convergent correlation and regression results and remained stable after missing‐data and hospital sensitivity analyses. Moral sensitivity estimates were directionally more variable across contextual sensitivity checks, reinforcing the need for larger multisite studies capable of evaluating organizational effect modification without sparse hospital‐specific samples.

### 4.1. Strengths and Limitations

Strengths include random selection from eligible sampling frames across four hospitals, inclusion of several cardiac‐unit types and shifts, an a priori sample‐size calculation for the primary correlation objective, explicit analytic denominators, confidence intervals and effect sizes, multiplicity adjustment for exploratory comparisons, and extensive robustness assessment. Sensitivity analyses addressed incomplete data, unequal hospital participation, alternative outcome transformations, and influential observations. Hospital fixed effects and leave‐one‐hospital‐out analyses directly examined whether the dominant hospital explained the principal finding.

Several limitations remain. First, the cross‐sectional design precludes temporal and causal inference: Emotional exhaustion may precede lower happiness, lower happiness may increase vulnerability to exhaustion, or both may reflect shared organizational or individual conditions. Second, all principal constructs were self‐reported and may be affected by response style, social desirability, and common‐method variance. Third, internal consistency of the eight‐item happiness measure was modest (alpha = 0.652), which may reduce measurement precision. Fourth, complete‐case requirements reduced the adjusted model to 181 participants. Missingness was associated with observed characteristics; missing completely at random was not assumed, and although imputation and weighting analyses yielded similar emotional exhaustion estimates, residual bias from unobserved determinants of missingness cannot be excluded. Fifth, one hospital contributed 69.5% of participants. Fixed‐effect and leave‐one‐hospital‐out analyses indicated that the principal emotional exhaustion finding was not driven by that hospital, but only four hospitals participated, limiting reliable higher‐level modeling and generalizability. Sixth, the a priori sample‐size calculation addressed the primary correlation objective rather than the secondary multivariable model, so small adjusted associations were estimated with less precision. Finally, moral distress, ethical climate, staffing, workload, leadership style, organizational support, job satisfaction, resilience, and social support were not measured, leaving plausible mechanisms and residual confounding untested.

## 5. Implications for Nursing Management

For nursing management, emotional exhaustion can be treated as a potential workforce‐level signal of strain rather than solely as an individual coping problem. This study does not establish that reducing exhaustion will increase happiness; the appropriate implication is to identify and prospectively evaluate organizational conditions that may accompany both emotional depletion and well‐being. In cardiac services, confidential workforce monitoring could combine brief exhaustion and well‐being indicators with unit‐level information on staffing, workload, overtime, shift patterns, missed or delayed breaks, sickness absence, turnover signals, and access to recovery opportunities. Such information should support organizational review rather than individual performance appraisal. Potential responses for prospective evaluation include workload and staffing review, reducing avoidable overtime or repeatedly adverse scheduling patterns, protecting rest and meal breaks, strengthening supportive supervision and peer support, and ensuring clear access to confidential employee or psychological support.

The moral sensitivity findings point to a complementary priority. Ethical awareness should be preserved rather than treated as a problem to be reduced. Managers can examine whether nurses have practical and psychologically safe mechanisms for raising and resolving ethical concerns, including responsive supervision, accessible ethics consultation, structured interdisciplinary case reflection or debriefing, and escalation pathways for recurring ethical constraints. Taken together, the findings support a dual management approach: protecting nurses’ emotional resources while maintaining the ethical capacities required for patient‐centered nursing practice. Longitudinal and intervention‐based evaluation is needed to determine whether changes in work resources, recovery opportunities, and ethical support are followed by changes in emotional exhaustion and well‐being.

## 6. Conclusions

Among nurses working in cardiac wards, emotional exhaustion showed the stronger and more consistent inverse association with happiness. The adjusted association between total moral sensitivity and happiness was smaller and less precise, and its inferential status was more sensitive to analytic approach; moral sensitivity should therefore not be interpreted as either a harmful professional characteristic or a definitively unrelated factor. The findings support managerial attention to emotional depletion and the organizational conditions that may accompany it while preserving structures that enable ethical nursing practice. Longitudinal, multilevel, and intervention studies are needed to establish directionality and evaluate whether changes in work resources and ethical support are followed by changes in nurses’ well‐being [20, 21].

## Author Contributions

Seyed Ali Rasooli contributed to the conception and design of the study. Seyed Ali Rasooli and Safoura Dorri contributed to data collection. Seyed Ali Rasooli performed the data analysis. Seyed Ali Rasooli and Safoura Dorri interpreted the findings. Safoura Dorri drafted the initial manuscript. Both authors critically revised the manuscript for important intellectual content.

## Funding

This research received no specific grant from any funding agency in the public, commercial, or not‐for‐profit sectors.

## Disclosure

All authors approved the final version and agreed to be accountable for all aspects of the work.

## Ethics Statement

The study was approved by the Ethics Committee of Isfahan University of Medical Sciences (IR.MUI.NUREMA.REC.1403.074). Written informed consent was obtained from all participants.

## Conflicts of Interest

The authors declare no conflicts of interest.

## Supporting Information

Additional supporting information can be found online in the Supporting Information section.

## Supporting information


**Supporting Information 1** Supporting Information 1: Tables S1–S8. Supporting Table S1. Additional sociodemographic, lifestyle, and work‐related characteristics of participants. Supporting Table S2. Descriptive statistics and percentage‐of‐maximum‐possible scores for moral sensitivity subscales and distribution of total moral sensitivity levels. Supporting Table S3. Exploratory correlations between happiness and moral sensitivity subscales. Supporting Table S4. Exploratory bivariate associations between happiness and sociodemographic, occupational, lifestyle, and work‐related characteristics. Supporting Table S5. Sensitivity analyses for nominal subgroup findings involving very small categories. Supporting Table S6. Expanded diagnostics and robustness checks for the multivariable linear regression predicting happiness. Supporting Table S7. Multivariable robustness and hospital influence analyses. Supporting Table S8. Missing‐data patterns and complete‐case attrition.

**Supporting Information 2** JNM_Revised_STROBE_Checklist

## Data Availability

The study dataset is not publicly available. Requests for access to de‐identified data may be directed to the corresponding author and will be considered subject to institutional ethical requirements and any applicable third‐party instrument restrictions.
