# Supplementary material for: Emotional Exhaustion, Moral Sensitivity, and Happiness Among Nurses in Cardiac Wards: A Multicenter Cross‐Sectional Study
Source: J Nurs Manag. 2026 Oct 6;2026:6483265. doi: 10.1155/jonm/6483265 (PMC13639924; doi:10.1155/jonm/6483265)
Supplement: Supplementary file 1 [file JONM-2026-6483265-s001.docx]

**SUPPORTING INFORMATION**

**Supporting Tables S1-S8**

Manuscript title

**Emotional Exhaustion, Moral Sensitivity, and Happiness Among Nurses in Cardiac Wards: A Multicentre Cross-Sectional Study**

Journal: Journal of Nursing Management

**Contents**

| **Table** | **Content** |
| --- | --- |
| S1 | Additional sociodemographic, lifestyle, and work-related characteristics |
| S2 | Moral sensitivity subscale scores, POMP values, and total-score categories |
| S3 | Exploratory correlations between happiness and moral sensitivity subscales |
| S4 | Exploratory bivariate associations with family-wise Holm adjustment |
| S5 | Small-cell sensitivity analyses for nominal subgroup findings |
| S6 | Expanded regression diagnostics and transformation checks |
| S7 | Multivariable robustness and hospital influence analyses |
| S8 | Missing-data patterns and complete-case attrition |

This file contains editable supporting tables cited in the main manuscript. It contains no direct participant identifiers and is intended for online publication as Supporting Information.

## **Supporting Table S1. Additional sociodemographic, lifestyle, and work-related characteristics of participants**

| **Characteristic** | **Category** | **n/valid N (%)** | **Missing, n** |
| --- | --- | --- | --- |
| Housing status | Rented | 86/217 (39.6) | 3 |
| Housing status | Owned | 131/217 (60.4) | — |
| Second job | No | 153/214 (71.5) | 6 |
| Second job | Yes | 61/214 (28.5) | — |
| Leisure time | <1 hour/week | 70/218 (32.1) | 2 |
| Leisure time | 2–4 hours/week | 106/218 (48.6) | — |
| Leisure time | >4 hours/week | 42/218 (19.3) | — |
| Satisfaction with health status | High | 72/217 (33.2) | 3 |
| Satisfaction with health status | Moderate | 130/217 (59.9) | — |
| Satisfaction with health status | Low | 15/217 (6.9) | — |
| Interest in the nursing profession | High | 34/218 (15.6) | 2 |
| Interest in the nursing profession | Moderate | 128/218 (58.7) | — |
| Interest in the nursing profession | Low | 56/218 (25.7) | — |
| Interest in the work environment | High | 25/220 (11.4) | 0 |
| Interest in the work environment | Moderate | 128/220 (58.2) | — |
| Interest in the work environment | Low | 67/220 (30.5) | — |

**Note.** Data are presented as n/valid N (%) unless otherwise indicated. Percentages were calculated using valid denominators after excluding missing values. An em dash indicates that the missing count is reported on the first category row for the corresponding characteristic.

## **Supporting Table S2. Descriptive statistics and percentage-of-maximum-possible scores for moral sensitivity subscales and distribution of total moral sensitivity levels**

**Panel A. Moral sensitivity subscale scores**

| **Moral sensitivity subscale** | **Possible score range** | **n** | **Mean ± SD (95% CI)** | **Mean POMP, %** | **Median (IQR)** | **Observed range** | **Shapiro-Wilk p** |
| --- | --- | --- | --- | --- | --- | --- | --- |
| Respect for patient autonomy | 0–12 | 218 | 7.44 ± 2.13 (7.16 to 7.72) | 62.0 | 7 (6–9) | 3–12 | <0.001 |
| Awareness of communication with patients | 0–20 | 207 | 14.86 ± 3.48 (14.38 to 15.33) | 74.3 | 15 (13–17) | 0–20 | <0.001 |
| Professional knowledge | 0–8 | 216 | 3.25 ± 1.89 (3.00 to 3.51) | 40.6 | 3 (2–4) | 0–8 | <0.001 |
| Experiencing ethical problems and dilemmas | 0–12 | 220 | 7.75 ± 2.03 (7.48 to 8.02) | 64.6 | 8 (6–9) | 3–12 | <0.001 |
| Applying ethical concepts in ethical decision-making | 0–20 | 210 | 11.96 ± 3.35 (11.50 to 12.41) | 59.8 | 12 (10–15) | 3–20 | 0.002 |
| Honesty and benevolence | 0–28 | 216 | 15.30 ± 4.49 (14.70 to 15.90) | 54.6 | 15 (13–17) | 3–28 | <0.001 |

**Panel B. Distribution of moral sensitivity levels**

| **Moral sensitivity category** | **n/valid N (%)** | **n/total sample (%)** |
| --- | --- | --- |
| Low, <50 | 29/195 (14.9) | 29/220 (13.2) |
| Moderate, 50 to <75 | 140/195 (71.8) | 140/220 (63.6) |
| High, ≥75 | 26/195 (13.3) | 26/220 (11.8) |
| Missing | — | 25/220 (11.4) |

**Note.** CI, confidence interval; IQR, interquartile range; POMP, percentage of maximum possible; SD, standard deviation. POMP was calculated as [(observed score - minimum possible)/(maximum possible - minimum possible)] x 100 and is provided only to facilitate descriptive comparison across subscales with different score ranges. Raw validated subscale scores were retained for all statistical analyses. Cronbach’s alpha is reported for the total moral sensitivity scale rather than individual subscales.

## **Supporting Table S3. Exploratory correlations between happiness and moral sensitivity subscales**

| **Relationship** | **n** | **Pearson r** | **95% CI** | **Pearson p** | **Holm-adjusted Pearson p** | **Spearman rho** | **Spearman p** | **Holm-adjusted Spearman p** |
| --- | --- | --- | --- | --- | --- | --- | --- | --- |
| Happiness × Respect for patient autonomy | 214 | -0.074 | -0.206 to 0.061 | 0.283 | 0.566 | -0.085 | 0.217 | 0.434 |
| Happiness × Awareness of communication with patients | 203 | 0.002 | -0.136 to 0.139 | 0.981 | 0.981 | -0.056 | 0.431 | 0.434 |
| Happiness × Professional knowledge | 212 | -0.137 | -0.267 to -0.003 | 0.046 | 0.184 | -0.117 | 0.090 | 0.270 |
| Happiness × Experiencing ethical problems and dilemmas | 216 | -0.163 | -0.290 to -0.030 | 0.017 | 0.085 | -0.157 | 0.021 | 0.105 |
| Happiness × Applying ethical concepts in ethical decision-making | 206 | -0.116 | -0.249 to 0.021 | 0.096 | 0.288 | -0.142 | 0.042 | 0.168 |
| Happiness × Honesty and benevolence | 212 | -0.338 | -0.452 to -0.213 | <0.001 | <0.006 | -0.378 | <0.001 | <0.006 |

**Note.** CI, confidence interval. These subscale-level correlations were exploratory secondary analyses. Holm-adjusted p values were calculated separately for Pearson and Spearman analyses to control the family-wise error rate across the six predefined instrument subscales. Pearson correlations were the primary estimates and Spearman rank correlations were sensitivity analyses. After Holm adjustment, honesty and benevolence was the only subscale for which the association with happiness remained statistically supported; this finding is hypothesis-generating.

## **Supporting Table S4. Exploratory bivariate associations between happiness and sociodemographic, occupational, lifestyle, and work-related characteristics**

**Panel A. Sociodemographic and occupational characteristics**

| **Characteristic** | **Category** | **n** | **Happiness, mean ± SD** | **Test** | **Statistic** | **p** | **Effect size** | **Holm-adjusted p** |
| --- | --- | --- | --- | --- | --- | --- | --- | --- |
| Age group, years | 20–29 | 84 | 34.14 ± 5.72 | One-way ANOVA | 2.36 | 0.073 | η² = 0.033 | 0.898 |
|  | 30–39 | 90 | 34.07 ± 5.78 |  |  |  |  |  |
|  | 40–49 | 33 | 35.94 ± 6.45 |  |  |  |  |  |
|  | ≥50 | 4 | 40.50 ± 0.58 |  |  |  |  |  |
| Sex | Male | 59 | 35.80 ± 5.03 | Independent t-test | 2.05 | 0.042 | Cohen’s d = 0.313 | 0.581 |
| Sex | Female | 157 | 33.99 ± 6.03 |  |  |  |  | 0.581 |
| Marital status | Married | 133 | 34.97 ± 6.01 | Welch t-test | 1.60 | 0.111 | Cohen’s d = 0.219 | 0.910 |
| Marital status | Single | 83 | 33.70 ± 5.45 |  |  |  |  | 0.910 |
| Education | Bachelor’s degree | 192 | 34.24 ± 5.67 | Independent t-test | -1.70 | 0.091 | Cohen’s d = -0.368 | 0.910 |
| Education | Master’s degree | 24 | 36.38 ± 6.77 |  |  |  |  | 0.910 |
| Employment status | Obligatory service | 29 | 34.28 ± 4.88 | One-way ANOVA | 1.65 | 0.195 | η² = 0.015 | 1.000 |
| Employment status | Contractual/temporary | 119 | 35.08 ± 5.55 |  |  |  |  | 1.000 |
| Employment status | Permanent | 66 | 33.47 ± 6.65 |  |  |  |  | 1.000 |
| Clinical work experience, months | <60 | 85 | 34.08 ± 5.63 | One-way ANOVA | 2.48 | 0.033 | η² = 0.056 | 0.492 |
| Clinical work experience, months | 60–119 | 66 | 34.73 ± 6.23 |  |  |  |  | 0.492 |
| Clinical work experience, months | 120–179 | 35 | 32.91 ± 5.14 |  |  |  |  | 0.492 |
| Clinical work experience, months | 180–239 | 15 | 35.67 ± 5.64 |  |  |  |  | 0.492 |
| Clinical work experience, months | 240–312 | 9 | 36.22 ± 6.57 |  |  |  |  | 0.492 |
| Clinical work experience, months | >312 | 6 | 41.00 ± 0.89 |  |  |  |  | 0.492 |
| Hospital | Alzahra | 26 | 35.00 ± 4.47 | Welch ANOVA | 2.50 | 0.076 | η² = 0.030 | 0.898 |
| Hospital | Feiz | 10 | 37.30 ± 4.32 |  |  |  |  | 0.898 |
| Hospital | Noor | 31 | 36.06 ± 7.08 |  |  |  |  | 0.898 |
| Hospital | Chamran | 149 | 33.87 ± 5.75 |  |  |  |  | 0.898 |
| Cardiac ward type | Internal cardiac ward | 74 | 35.26 ± 5.79 | One-way ANOVA | 1.04 | 0.356 | η² = 0.010 | 1.000 |
| Cardiac ward type | Cardiac surgery ward | 50 | 34.26 ± 5.09 |  |  |  |  | 1.000 |
| Cardiac ward type | Special cardiac care units | 92 | 33.98 ± 6.20 |  |  |  |  | 1.000 |
| Monthly income, million Tomans | <10 | 18 | 34.72 ± 3.97 | Welch ANOVA | 7.12 | 0.004 | η² = 0.044 | 0.078 |
| Monthly income, million Tomans | 10–15 | 185 | 34.22 ± 5.82 |  |  |  |  | 0.078 |
| Monthly income, million Tomans | 15–20 | 6 | 41.67 ± 3.61 |  |  |  |  | 0.078 |
| Monthly income, million Tomans | >20 | 7 | 34.71 ± 8.10 |  |  |  |  | 0.078 |

**Panel B. Lifestyle and work-related characteristics**

| **Characteristic** | **Category** | **n** | **Happiness, mean ± SD** | **Test** | **Statistic** | **p** | **Effect size** | **Holm-adjusted p** |
| --- | --- | --- | --- | --- | --- | --- | --- | --- |
| Housing status | Rented | 84 | 35.39 ± 5.62 | Independent t-test | 1.83 | 0.069 | Cohen’s d = 0.256 | 0.898 |
| Housing status | Owned | 129 | 33.90 ± 5.96 |  |  |  |  | 0.898 |
| Second job | No | 149 | 34.24 ± 5.67 | Independent t-test | -0.68 | 0.495 | Cohen’s d = -0.104 | 1.000 |
| Second job | Yes | 61 | 34.85 ± 6.39 |  |  |  |  | 1.000 |
| Physical activity | Yes | 77 | 35.88 ± 5.49 | Independent t-test | 2.67 | 0.008 | Cohen’s d = 0.379 | 0.130 |
| Physical activity | No | 139 | 33.71 ± 5.87 |  |  |  |  | 0.130 |
| Leisure time | <1 hour/week | 68 | 33.41 ± 6.74 | Welch ANOVA | 1.84 | 0.164 | η² = 0.019 | 1.000 |
| Leisure time | 2–4 hours/week | 104 | 34.75 ± 5.32 |  |  |  |  | 1.000 |
| Leisure time | >4 hours/week | 42 | 35.62 ± 5.37 |  |  |  |  | 1.000 |
| Night shifts | 0–4 | 62 | 34.15 ± 5.49 | Welch ANOVA | 0.28 | 0.755 | η² = 0.002 | 1.000 |
| Night shifts | 4–8 | 73 | 34.75 ± 4.82 |  |  |  |  | 1.000 |
| Night shifts | >8 | 79 | 34.23 ± 6.73 |  |  |  |  | 1.000 |
| Satisfaction with health status | High | 72 | 35.11 ± 5.91 | One-way ANOVA | 1.14 | 0.322 | η² = 0.011 | 1.000 |
| Satisfaction with health status | Moderate | 126 | 34.42 ± 5.86 |  |  |  |  | 1.000 |
| Satisfaction with health status | Low | 15 | 32.67 ± 5.37 |  |  |  |  | 1.000 |
| Interest in the nursing profession | High | 34 | 36.50 ± 5.41 | One-way ANOVA | 2.41 | 0.093 | η² = 0.022 | 0.910 |
| Interest in the nursing profession | Moderate | 126 | 34.18 ± 5.88 |  |  |  |  | 0.910 |
| Interest in the nursing profession | Low | 54 | 34.00 ± 5.86 |  |  |  |  | 0.910 |
| Interest in the work environment | High | 25 | 36.28 ± 6.41 | One-way ANOVA | 1.38 | 0.253 | η² = 0.013 | 1.000 |
| Interest in the work environment | Moderate | 126 | 34.32 ± 5.38 |  |  |  |  | 1.000 |
| Interest in the work environment | Low | 65 | 34.11 ± 6.35 |  |  |  |  | 1.000 |
| Conflict with others | High | 18 | 36.33 ± 5.65 | One-way ANOVA | 5.27 | 0.006 | η² = 0.048 | 0.100 |
| Conflict with others | Moderate | 72 | 32.79 ± 5.19 |  |  |  |  | 0.100 |
| Conflict with others | Low | 123 | 35.28 ± 6.04 |  |  |  |  | 0.100 |

**Note.** SD, standard deviation. Between-group analyses were exploratory. Test choice used conventional or Welch alternatives according to variance diagnostics. Holm-adjusted p values control the family-wise error rate across all 18 omnibus comparisons in this table. No omnibus comparison remained statistically significant after family-wise adjustment. Effect sizes are Cohen’s d for two-group comparisons and eta-squared for multi-group comparisons. Findings involving very small categories should be interpreted particularly cautiously.

## **Supporting Table S5. Sensitivity analyses for nominal subgroup findings involving very small categories**

| **Characteristic** | **Original omnibus p** | **Sensitivity analysis** | **Sensitivity p** |
| --- | --- | --- | --- |
| Clinical work experience | 0.033 | Exclude >312 months category (n=6) | 0.369 |
| Monthly income | 0.004 | Exclude 15-20 million Tomans category (n=6) | 0.885 |

**Note.** These post hoc sensitivity analyses were conducted solely to evaluate the stability of nominal exploratory findings involving very small subgroup cells. They were not used to redefine categories or support confirmatory subgroup conclusions. The experience analysis used one-way ANOVA after exclusion of the >312-month group; the income analysis used Welch ANOVA after exclusion of the 15-20-million-Toman group.

## **Supporting Table S6. Expanded diagnostics and robustness checks for the multivariable linear regression predicting happiness**

| **Diagnostic test or index** | **Value** | **p** |
| --- | --- | --- |
| Durbin-Watson statistic | 1.814 | — |
| Shapiro-Wilk residual normality | 0.971 | <0.001 |
| Residual skewness | -0.527 | — |
| Residual excess kurtosis | 0.134 | — |
| Breusch-Pagan LM test | 15.371 | 0.052 |
| Breusch-Pagan F test | 1.995 | 0.050 |
| Variance inflation factor range | 1.03-4.05 | — |
| Maximum \|externally studentized residual\| | 2.64 | — |
| Maximum Cook's distance | 0.043 | — |

**Note.** HC3 heteroscedasticity-consistent standard errors were used for primary regression inference because variance diagnostics were borderline and to reduce sensitivity to leverage. HC3 was not interpreted as a correction for residual non-normality. Square-root and logarithmic transformations increased the departure from residual normality; a Box-Cox transformation improved normality but increased evidence of heteroscedasticity and reduced coefficient interpretability. No transformation provided a consistent overall diagnostic advantage, so happiness was retained on its original 8-48 scale. Complete-case model n=181.

## **Supporting Table S7. Multivariable robustness and hospital influence analyses**

**Panel A. Primary and sensitivity models**

| **Analysis** | **Analytic n** | **Emotional exhaustion B (95% CI)** | **Moral sensitivity B (95% CI)** |
| --- | --- | --- | --- |
| Primary complete-case HC3 | 181 | -0.184 (-0.262 to -0.107) | -0.069 (-0.140 to 0.002) |
| Hospital fixed effects | 181 | -0.180 (-0.258 to -0.102) | -0.069 (-0.146 to 0.008) |
| Item-level multiple imputation, m=50 | 220 | -0.191 (-0.265 to -0.117) | -0.061 (-0.126 to 0.004) |
| Item-level MI + hospital fixed effects | 220 | -0.186 (-0.260 to -0.111) | -0.061 (-0.128 to 0.007) |
| Inverse-probability weighting | 181 weighted | -0.189 (-0.269 to -0.109) | -0.073 (-0.144 to -0.001) |

**Panel B. Leave-one-hospital-out influence analysis for emotional exhaustion**

| **Analysis** | **n** | **Emotional exhaustion B (95% CI)** |
| --- | --- | --- |
| Hospital A excluded | 164 | -0.178 (-0.260 to -0.096) |
| Hospital B excluded | 173 | -0.177 (-0.257 to -0.096) |
| Hospital C excluded | 152 | -0.193 (-0.272 to -0.114) |
| Hospital D (largest hospital) excluded | 54 | -0.207 (-0.341 to -0.073) |

**Note.** B, unstandardized coefficient; CI, confidence interval; HC3, heteroscedasticity-consistent standard error type 3; MI, multiple imputation. Hospital D contributed 69.5% of the full sample. Leave-one-hospital-out models are influence analyses, not hospital-specific effect estimates. Because only four hospitals were available and some hospital-specific complete-case samples were small, formal hospital-specific effect modification was not treated as a stable inferential analysis.

## **Supporting Table S8. Missing-data patterns and complete-case attrition**

**Panel A. Variables responsible for exclusion from the complete-case regression**

| **Variable** | **Missing n** | **% of total sample** |
| --- | --- | --- |
| Total moral sensitivity | 25 | 11.4 |
| Age | 5 | 2.3 |
| Happiness | 4 | 1.8 |
| Interpersonal conflict | 3 | 1.4 |
| Emotional exhaustion | 2 | 0.9 |
| Total incomplete for regression | 39 | 17.7 |

**Panel B. Item-level missingness in the three principal scales**

| **Scale** | **Missing item responses** | **Missing item-cell rate, %** |
| --- | --- | --- |
| Happiness (8 items) | 6 of 1760 | 0.34 |
| Moral sensitivity (25 items) | 37 of 5500 | 0.67 |
| Emotional exhaustion (9 items) | 2 of 1980 | 0.10 |

**Panel C. Observed characteristics associated with complete-case status**

| **Comparison** | **Test** | **Statistic** | **p** |
| --- | --- | --- | --- |
| Hospital | Pearson chi-square | 7.879 (df=3) | 0.049 |
| Physical activity | Fisher exact | odds ratio=2.676 | 0.006 |
| Moral-sensitivity missingness by hospital | Pearson chi-square | 11.226 (df=3) | 0.011 |

**Note.** Each of the 39 participants excluded from the complete-case regression was missing exactly one model variable. Missingness was therefore sparse at the individual-item level but accumulated across variables under complete-case analysis. Because missingness was associated with observed hospital and participant characteristics, missing completely at random was not assumed. Multiple-imputation analyses were interpreted under a missing-at-random assumption conditional on observed variables; residual missing-not-at-random bias cannot be excluded.

Multiple-imputation specification. The item-level sensitivity analysis generated 50 imputed datasets using a chained donor-based predictive mean matching procedure. Missing scale items were imputed conditional on remaining items from the corresponding scale, the other principal study measures, age, interpersonal conflict, and fully observed demographic/work auxiliaries (sex, physical activity, income, hospital, marital status, education, ward, and interest in the work environment). Age and interpersonal-conflict values missing from the regression model were also updated within the donor-based procedure. Five chained updating sweeps and five nearest predictive donors were used per imputed dataset. Scale totals were recalculated within each completed dataset, HC3 regression estimates were obtained, and estimates were combined using Rubin’s rules. This sensitivity analysis assumes missing at random conditional on the included observed variables; residual missing-not-at-random bias cannot be excluded.
