# Supplementary material for: Emotional Exhaustion, Moral Sensitivity, and Happiness Among Nurses in Cardiac Wards: A Multicenter Cross‐Sectional Study
Source: J Nurs Manag. 2026 Oct 6;2026:6483265. doi: 10.1155/jonm/6483265 (PMC13639924; doi:10.1155/jonm/6483265)
Supplement: Supplementary file 2 [file JONM-2026-6483265-s002.docx]

**STROBE Statement—Checklist for Cross-Sectional Studies**

Manuscript: Emotional Exhaustion, Moral Sensitivity, and Happiness Among Nurses in Cardiac Wards: A Multicentre Cross-Sectional Study

Reporting guideline statement in manuscript: Reporting was guided by the STROBE recommendations for cross-sectional studies.

| **Section/topic** | **Item** | **STROBE recommendation** | **Where reported** |
| --- | --- | --- | --- |
| Title and abstract | 1(a) | Indicate the study design with a commonly used term in the title or abstract. | Main manuscript: Title and Abstract |
| Title and abstract | 1(b) | Provide an informative and balanced summary of what was done and found. | Main manuscript: Abstract |
| Background/rationale | 2 | Explain the scientific background and rationale. | Main manuscript: Background |
| Objectives | 3 | State specific objectives, including prespecified hypotheses where relevant. | Main manuscript: Background, final paragraph |
| Study design | 4 | Present key elements of the study design early in the paper. | Main manuscript: Methods—Study design and setting |
| Setting | 5 | Describe setting, locations, and relevant dates, including periods of recruitment and data collection. | Main manuscript: Methods—Study design and setting |
| Participants | 6(a) | Give eligibility criteria and the sources and methods of participant selection. | Main manuscript: Methods—Participants, sample size, and sampling |
| Variables | 7 | Clearly define outcomes, exposures, predictors, confounders, and effect modifiers; provide diagnostic criteria where relevant. | Main manuscript: Methods—Measures and Statistical analysis |
| Data sources/measurement | 8 | For each variable, give sources of data and details of assessment methods; describe comparability across groups when relevant. | Main manuscript: Methods—Measures |
| Bias | 9 | Describe efforts to address potential sources of bias. | Main manuscript: Methods—sampling, measurement, robust/sensitivity analyses; Strengths and Limitations |
| Study size | 10 | Explain how the study size was determined. | Main manuscript: Methods—Participants, sample size, and sampling |
| Quantitative variables | 11 | Explain how quantitative variables were handled; describe groupings and reasons, when applicable. | Main manuscript: Methods—Statistical analysis; Tables 1 and 4 |
| Statistical methods | 12(a) | Describe all statistical methods, including methods used to control for confounding. | Main manuscript: Methods—Statistical analysis |
| Statistical methods | 12(b) | Describe methods used to examine subgroups and interactions. | Main manuscript: Methods—Statistical analysis; Supporting Tables S3-S5 |
| Statistical methods | 12(c) | Explain how missing data were addressed. | Main manuscript: Methods—Statistical analysis; Results—missing data; Supporting Table S8 |
| Statistical methods | 12(d) | If applicable, describe analytical methods that account for the sampling strategy. | Main manuscript: Methods—sampling strategy and hospital fixed-effects sensitivity; Supporting Table S7 |
| Statistical methods | 12(e) | Describe sensitivity analyses. | Main manuscript: Methods—Statistical analysis; Supporting Tables S5-S8 |
| Participants | 13(a) | Report numbers at each stage of the study, such as potentially eligible, assessed, included, and analysed. | Main manuscript: Results—Participant characteristics and missing data; Supporting Table S8. Exact pre-enrolment screening counts unavailable. |
| Participants | 13(b) | Give reasons for non-participation at each stage. | Pre-enrolment criterion-specific counts were not retained; this is stated explicitly in Methods and the response letter. Analysis-specific exclusions are fully reported. |
| Participants | 13(c) | Consider use of a flow diagram. | No flow diagram included because pre-enrolment stage counts were not available; analytic flow is reported narratively and in Supporting Table S8. |
| Descriptive data | 14(a) | Give participant characteristics and information on exposures and potential confounders. | Main manuscript: Results—Participant characteristics; Table 1; Supporting Table S1 |
| Descriptive data | 14(b) | Indicate the number of participants with missing data for each variable of interest. | Main manuscript: Results—missing data; Tables 1-4; Supporting Table S8 |
| Descriptive data | 14(c) | Summarize follow-up time, if relevant. | Not applicable to the cross-sectional design. |
| Outcome data | 15 | Report numbers of outcome events or summary measures. | Main manuscript: Results—Study variables and reliability; Table 2 |
| Main results | 16(a) | Give unadjusted and adjusted estimates and their precision; identify adjusted confounders and explain their inclusion. | Main manuscript: Results—Correlations and Multivariable analysis; Tables 3-4; Supporting Table S7 |
| Main results | 16(b) | Report category boundaries when continuous variables were categorized. | Table 1; Methods—Statistical analysis |
| Main results | 16(c) | Translate relative risk estimates into absolute risk when relevant. | Not applicable; continuous outcome and linear associations. |
| Other analyses | 17 | Report other analyses, including subgroup, interaction, and sensitivity analyses. | Main manuscript: Results—Exploratory group comparisons and Multivariable analysis; Supporting Tables S3-S8 |
| Key results | 18 | Summarize key results with reference to study objectives. | Main manuscript: Discussion, opening paragraph |
| Limitations | 19 | Discuss study limitations, including potential bias and imprecision, with direction and magnitude where possible. | Main manuscript: Strengths and Limitations |
| Interpretation | 20 | Provide a cautious overall interpretation considering objectives, limitations, multiple analyses, and other evidence. | Main manuscript: Discussion and Conclusions |
| Generalisability | 21 | Discuss the generalisability of the study results. | Main manuscript: Strengths and Limitations |
| Funding | 22 | Give the source of funding and the role of funders. | Separate title page: Funding |

Note: Eligibility screening preceded random selection. Exact counts screened or excluded before formation of the eligible sampling frames were not retained and are therefore not retrospectively estimated. All 220 enrolled participants are accounted for, and analysis-specific missingness is reported in the manuscript and Supporting Table S8.
